# Parallel but independent reduction of emotional awareness and corpus callosum connectivity in older age

**DOI:** 10.1371/journal.pone.0209915

**Published:** 2018-12-31

**Authors:** Martine Skumlien, Donatas Sederevicius, Anders M. Fjell, Kristine B. Walhovd, René Westerhausen

**Affiliations:** 1 Center for Lifespan Changes in Brain and Cognition (LCBC), Department of Psychology, University of Oslo, Oslo, Norway; 2 Department of Radiology and Nuclear Medicine, Oslo University Hospital, Oslo, Norway; 3 Department of Biological and Medical Psychology, University of Bergen, Bergen, Norway; Emory University, UNITED STATES

## Abstract

Differential functional specialization of the left and right hemispheres for linguistic and emotional functions, respectively, suggest that interhemispheric communication via the corpus callosum is critical for emotional awareness. Accordingly, it has been hypothesized that the age-related decline in callosal connectivity mediates the frequently demonstrated reduction in emotional awareness in older age. The present study tests this hypothesis in a sample of 307 healthy individuals between 20–89 years using combined structural and diffusion-tensor magnetic resonance imaging (MRI) of the corpus callosum. As assumed, inter-hemispheric connectivity (midsagittal callosal area and thickness, as well as fractional anisotropy, FA) and emotional awareness (i.e., increase in externally-oriented thinking, EOT; assessed with the Toronto Alexithymia Scale, TAS-20) were found to be reduced in older (> 60 years) compared to younger participants. Furthermore, relating callosal measures to emotional awareness, FA in the genu of the corpus callosum was found to be negatively correlated with EOT in male participants. Thus, “stronger” structural connectivity (higher FA) was related with higher emotional awareness (lower EOT). However, a formal mediation analysis did not support the notion that age-related decline in emotional awareness is mediated by the corpus callosum. Thus, the observed reduction of emotional awareness and callosal connectivity in older age likely reflects parallel but not inter-dependent processes.

## Introduction

The concept *emotional awareness* summarizes an individual’s clarity about and attention to emotional experiences [1–3]. While the individual degree of emotional awareness is considered a personality trait [4, 5], substantial inter-individual differences have been reported which also have been found to be age-related [6, 7]. While studies on the content of emotional experiences indicate an improvement in emotional well-being into middle and old age [8, 9], emotional awareness appears to decline [10–14]. For example, using self-report measures, a substantial reduction in emotional awareness in older age can be observed [15–18].

While this age-associated effect might to some degree reflect changes in the cognitive strategies in dealing with emotions, a well-established line of research suggests that individual difference in emotional awareness are related to alterations in brain anatomy (for a review see [19, 20]). In particular, one long-standing hypothesis originates from observations in corpus callosotomy patients, as these patients often express considerable deficits in awareness of own emotions, so-called alexithymia, suggesting a role of the corpus callosum for the ability to verbally identify, interpret, and communicate emotions [21–24]. This suggestion is embedded in the assumption of a left-right dichotomy of hemispheric specialization, that is, that the left hemisphere is specialized for linguistic functions [25–27] while the right hemisphere is thought to be specialized for the processing of emotions [28, 29, 30], so that the transfer of emotional information from the right hemisphere to the verbally competent left hemisphere would be crucial for the identification and communication of emotions [20]. Although the assumption of exclusive right hemisphere emotion processing might be questioned, and alternative models have been suggested [31, 32], the above outlined “callosal-relay hypothesis” has received substantial support. That is, besides the observations in callosotomy patients, also in studies on patients with congenital callosal agenesis [33–35] or in multiple sclerosis [36] and schizophrenia patients [7] with callosal pathology, correlations between anatomical callosal variability and emotional awareness have been reported. Habib et al. [36] found a smaller posterior corpus callosum to co-occur with a lower level of emotional awareness in multiple sclerosis patients. Kubota et al. [7], using diffusion-tensor imaging (DTI), revealed negative correlations between fractional anisotropy (FA) in the truncus/splenium of the corpus callosum and emotional awareness in schizophrenia patents. Thus, as predicted by the callosal-relay hypothesis, both studies suggest that stronger callosal connectivity is associated with higher emotional awareness. Unfortunately, both studies fail to report associations in healthy control samples so that it cannot be excluded that the reported effects are at least partly driven by pathological alterations and do not effect general structure-function associations. At the same time, further indirect evidence stems from a series of studies showing a link between emotional cognition and measures of callosal transfer efficacy also in healthy individuals. Using electroencephalographic recordings, Ten Houten et al. [37] found reduced interhemispheric coherence of neuronal activity between homologous scalp locations in the alpha band (i.e., less efficient interhemispheric communication) to be related to increased alexithymia-like symptoms both in patients and controls. Furthermore, inter-hemispheric transfer properties assessed using the finger localization task [38–40], transcranial magnetic stimulation [41], or visual half field paradigms [36, 42, 43] show positive associations of transfer efficacy and emotional awareness.

Older age, on the other hand, is related to an ongoing reduction in inter-hemispheric structural and functional connectivity. Midsagittal callosal area decreases with advancing age [44–46], and DTI studies show an accompanying reduction in FA and increase in mean diffusivity [47, 48]. These changes likely reflect a reduction in the number of callosal axons as well as progressing alterations in axon myelination as demonstrated in histological studies on humans [49] and lower primates [50], alterations which appear to be especially pronounced above the age of 60 years [51]. Also, measures of functional hemispheric interaction reveal a slowing of inter-hemispheric transfer time and integration efficiency with advancing age [52–55].

Taken together, given the suggested relevance of the corpus callosum for emotional awareness, it is tempting to speculate that the known reduction of inter-hemispheric connectivity with age also contributes to the reduction in emotional awareness in older age. The aim of the present study was to test this “callosal-mediation hypothesis” in a sample of 307 individuals, comprising men and women aged 20–89 years. Hemispheric structural connectivity was assessed using absolute and relative (to brain size) mid-sagittal callosal area, and callosal thickness, as obtained from structural MRI, as well as fractional anisotropy (FA) determined from diffusion-tensor imaging (DTI). Emotional awareness was assessed with the Toronto-Alexithymia Scale (TAS-20) [56, 57], including the three subscale Difficulties Identifying Feelings (DIF), Difficulties Describing Feelings (DDF), and Externally Oriented Thinking (EOT). Beyond the primary aim the present study also allowed to re-examine the “callosal-relay hypothesis” of emotional awareness by attempting to replicate previous correlations between structural callosal variability and measures of emotional awareness [36].

## Material and methods

### Participants

A total of 370 healthy right-handed participants, representing the third wave of the “Cognition and Plasticity through the Lifespan” project [58], were included in the present study. However, participants who lacked valid MRI/DTI-data and/or data on more than one TAS-20 question were excluded (*n =* 63). The resulting sample consisted of 307 participants; 94 male (30.6%) and 213 female (69.4%). The age ranged from 20.5 to 89.4 years (mean age ± standard deviation: 45.2 ± 17.7 years).

Of note, the age distribution of the sample was bimodal, so that the sample was divided into three age subgroups for further analyses. Division into three groups was conducted under consideration of established developmental trajectories of the corpus callosum. A Young age group (*n =* 87; 71.2% female) encompassed all participants under 30 years as callosal maturation has been reported to continue into the late 20s (e.g., [48, 59]). A middle age group (*n* = 130; 73.8% female) was formed to range from 30 to 59.9 years, a period for which a continuous slow decline in callosal size and diffusion parameters can be observed in cross-sectional studies [44, 48, 60]. Participants above 60 years formed the Older group (*n* = 90, 61.1% female), covering an age range characterized by an accelerated decline in fiber density [51]. The differences in the sex distribution between the three age groups was not significant in a 2x3 Chi-Squared test (*χ*^*2*^(2) = 4.26, *p* = .12, Cramer’s *V* = 0.12). All participants gave written informed consent for participation in the project. The study was approved by regional ethic committee REK sør-øst (reference 2010/3407).

### Assessment of emotional awareness

Participants completed the 20 item TAS, a self-report measure designed to assess alexithymia symptoms [56, 57, 61] which, however, is routinely used to assess nonclinical variability in emotional awareness [2, 16, 62, 63]. Questions are answered on a 5-point Likert scale, and possible total scores range from 20 to 100. The total scale is divided into three subscales: Difficulties Identifying Feelings (DIF), Difficulties Describing Feelings (DDF), and Externally Oriented Thinking (EOT). The Norwegian version of the TAS-20 was a translation of the Swedish version [64]. The translation was checked against the original English version by a proficient English speaker. Scale analysis of the Norwegian version of the TAS-20 indicated satisfying internal consistency for the total scale and the three subscales, with a Cronbach’s alpha value of .83 for the total score, and .83, .79, and .68 for DIF, DDF, and EOT subscales, respectively. Of note, nine participants lacked data on one of the items. To avoid excluding these participants from the analysis, the missing value was replaced with the mean item value for the given participant on the respective subscale.

### Magnetic resonance imaging

MRI was performed using a 3 Tesla Siemens Skyra scanner with a 24-channel head coil at Oslo University Hospital. A T1-weighted MPRAGE sequence (repetition time, TR = 2400 ms; echo time, TE = 3.61 ms; inversion time, TI = 1000 ms; flip angle = 8 degrees) with 176 sagittal slices (thickness: 1 mm; 256×256 scan matrix; 256×256 mm^2^ field of view) was acquired (image resolution: 1×1×1 mm^3^). DTI was based on a diffusion-weighted (b-value = 1000 s/mm^2^) spin-echo echo planar imaging (EPI) sequence, measuring diffusion in 64 gradient directions (TR = 9300 ms, TE = 87 ms). The sequence also included two reference (b = 0) images. Each volume consisted of 70 sagittal slices (thickness: 2 mm, 128×130 scan matrix, 252 x 256 mm^2^ field of view matrix) and voxel size of 2.0×1.97×1.97 mm^3^.

### Corpus callosum measures

Pre-processing of the raw images was performed with routines written in MATLAB (MathWorks Inc. Natick, MA, USA). Raw T1-images were coregistered to a template with SPM12 routines (Wellcome Department of Cognitive Neurology, London, UK) using a rigid-body transformation (i.e., rotation and translation only) such that the area measures remained unaffected by the coregistration step. The images were then segmented to obtain white-matter images in native space, the mid-sagittal slice was selected, and the corpus callosum was identified. The resulting segmentations were then visually inspected and manual corrections were applied if necessary (e.g., if white matter voxels belonging to the fornix were fused with voxels belonging to the corpus callosum). The individual callosal segmentation mask was then rotated such that the imagined line connecting the tip of the rostrum (posterior-most voxel of the in-bend anterior half) and base of the splenium (ventral-most voxel in the posterior half) was horizontally oriented. Subregional area was then determined employing a frequently used subdivision approach [65] dividing the total mid-sagittal surface area into three subregions. That is, relative to its anterior-posterior extension, the corpus callosum was divided into “genu” (defined as the anterior third), “truncus” (middle third), and the “posterior third” (encompassing isthmus and splenium). The total number of voxels belonging to each region was extracted as a measure of mid-sagittal surface area (in mm^2^).

To assess the effects of variation in the proportionality of the corpus callosum relative to brain size, the ratio of midsagittal area to total intracranial volume (tIV) was additionally determined for each subregion. Using the T1-weighted images, tIV was obtained applying the automatic segmentation routines (“tissue volumes” utility) provided with SPM12. This procedure defines tIV as total volume within the cranium, including all grey matter, white matter, and cerebrospinal fluid. As the dimensionality between area (2D) and tIV (3D) differs, ratios were calculated as area/tIV^0.667^. This adjustment allows for appropriate assessment of proportional similarity between corpora callosa in differently sized brains (for discussion see references [66, 67].

DTI analysis was performed based on the tract-based spatial statistics (TBSS) segmentation [68] as implemented using FSL (v5.0.10; Analysis Group, FMRIB, Oxford, UK) and the FMRIB's Diffusion Toolbox (FDT). Following the recommended approach individual diffusion-weighted images were corrected for eddy current-related distortions and for subject movement using the “eddy” routine, whereby “topup”-estimated fieldmaps were used to correct susceptibility-induced distortions. Individual FA images were then created using the “dtifit” function, and subjected to the TBSS analysis. That is, FA images were aligned in standard space by nonlinear registration, a mean FA skeleton was created (using an FA threshold of 0.2), and the individual FA data was projected onto this skeleton. To extract subregional FA values from the midline corpus callosum, midsagittal callosal masks of the three subregions were created based on the mean FA image. These were–analogously to the area subdivision described above–defined as callosal thirds along the anterior-posterior extension of the corpus callosum. Finally, the masks were utilized to extract the mean FA for genu, truncus, and posterior third from the individual skeleton images in standard space. This approach has previously yielded reliable FA measures for the corpus callosum, showing intra-class correlations between 0.79 for truncus and 0.82 for the genu FA measures across three measuring time points [69].

### Statistical analyses

The analysis strategy consistent of four steps. The first three analysis steps establish pairwise associations of (1) aging and emotional awareness, (2) aging and callosal measures; (3) callosal measures and emotional awareness. Analysis step (4) concerns the interaction of all three variables. Steps 1 to 3 are reflecting the first three conditions of a mediation analysis as suggested by Baron and Kenny [70], and are here established as analyses of (co-)variance (ANOVA/ANCOVA) to identify callosal segments and measures, TAS subscales, and conditions for which a mediation analysis is warranted. Analysis step 4 was then restricted to variables for which steps 1 to 3 indicate significant pairwise associations. Analysis step 4 represents a test of the callosal-mediation hypothesis of aging-related changes in emotional awareness, while analysis steps 3 and 4 taken together, represent tests of the callosal-relay hypothesis of emotional awareness.

Statistical analyses were done using IBM SPSS 25, whereby the mediation analysis was conducted using the PROCESS 3.1 macro [71]. Significance threshold of α = .05 was used for all tests, and significant results were followed by lower level post-hoc analyses where appropriate. For the mediation analysis, the estimation of the indirect effect was complemented with 95% confidence intervals (*CI*_95%_; determined with 5000 bootstrap samples) and additionally tested with the Aroian-Sobel test (quantpsy.org/sobel/sobel.htm). Effect sizes were expressed as proportion explained variance (*η*^*2*^) or Cohen’s *d* within the ANOVA designs, and as Partially Standardized Indirect Effect (ab_ps_) for the mediation analysis. Non-significant effects of interest were supplemented with sensitivity power analyses to determine the minimal population effect size (*η*_*min*_^2^ and partial R_min_^2^) that can be reliably excluded (settings: test power of *1-β* = 0.80, *α* = .05, degrees of freedom of the respective test). Power calculations were done using G*Power software [72].

## Results

### Aging and emotional awareness (analysis step 1)

**Fig 1:**
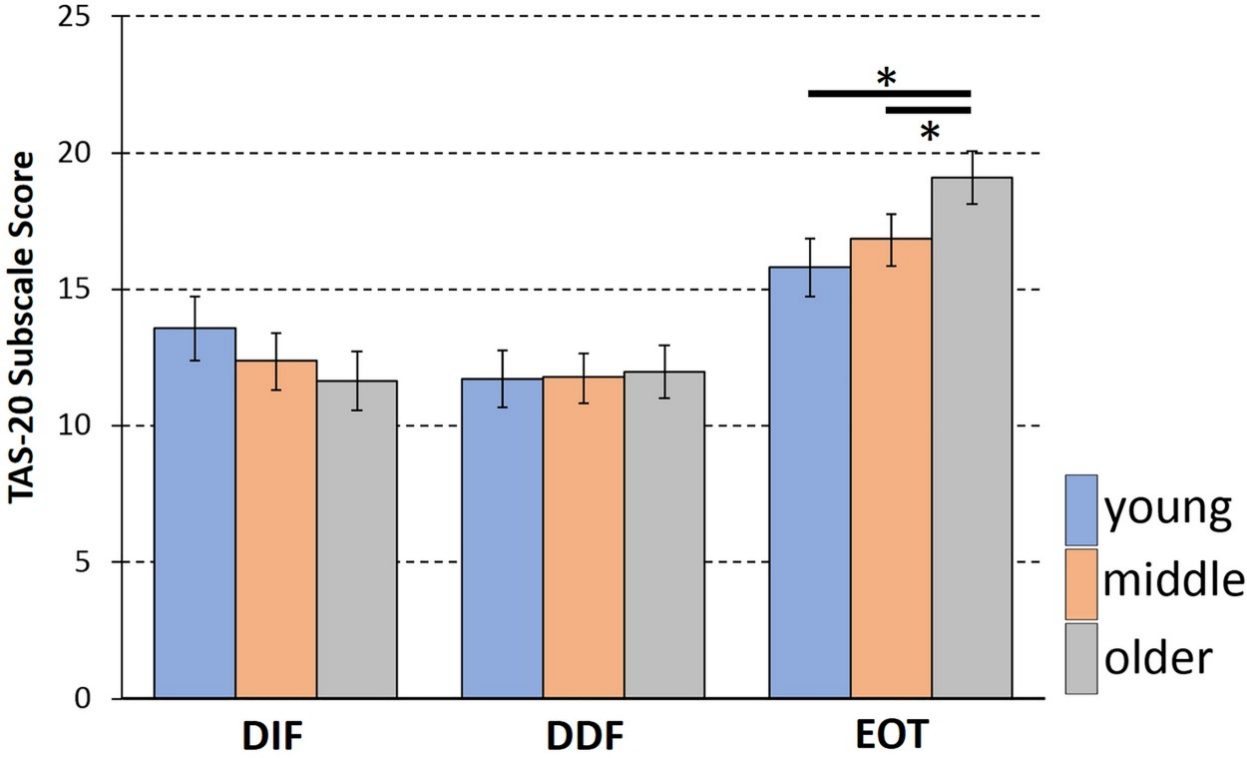
Aging and emotional awareness. Mean subscale scores for the three age groups, with error bars indicating the 95% confidence limits. A significant age group by subscale interaction (*η*^*2*^ = .03) was found whereby a significant Age group effect was only confirmed for the EOT subscale (right side of graph). Marked by asterisks (*) are the significant post-hoc pairwise comparisons for the EOT score. The older age group differed significantly from the two other groups, indicating an increase in externally oriented thinking, that is, a decrease in emotional awareness with advancing age.

Aging-related changes in emotional awareness were analysed using analysis of variance (ANOVA) including the between-subject factors age group (young [<30 years], middle [30–60 years], older [>60 years]; see participants section for details) and sex, as well as the repeated-measures factor subscale (DIF, DDF, EOT). While the main effect of age group was not significant, the omnibus ANOVA revealed a significant interaction of age group and subscale (*p* < .001, *η*^*2*^ = .03, Fig 1; test statistics see Table 1). Exploring the interaction, separate two-factorial ANOVAs (factors age group, sex) were calculated for each subscale. In the EOT post-hoc analysis, the main effect of age group was significant (*F*(2,301) = 10.760, *p* < .001, *η*^*2*^ = .06) with the older group having higher EOT scores than the two other groups (vs. young: *p* < .001, *d* = 0.67; vs. middle: *p* = .001, *d* = 0.45), and no significant difference between middle and young group (*p* = .145, *d* = 0.20). A comparable main effect was not found for DIF (*F*(2,301) = 2.803, *p* = .062, *η*^*2*^ = .018) or DDF subscales (*F*(2,301)<1, *p* = .927, *η*^*2*^ < .01). Furthermore, the omnibus ANOVA did not provide any indication that the above age effect was modulated by sex, as neither the age group by sex nor the three-way interaction yielded significance.

**Table 1:** Aging and emotional awareness. Results of the analysis of variance with the three factors age group (young, middle, older), sex (male/female), and subscale (within factor: DIF, DDF, EOT).

Effect	*F*	df_effect_	df_error_	*p*	*η*^*2*^
Age group	0.69	2	301	0.50	<0.01
Age Group x Subscale	9.51	4	602	**<0.001**	0.03
Age Group x Sex	0.09	2	301	0.92	<0.01
Age Group x Subscale x Sex	1.79	4	602	0.13	0.01
Sex ^a^	10.09	1	301	**<0.001**	0.02
Subscale x Sex ^b^	5.64	2	602	**0.003**	0.01
Scale ^c^	151.27	2	602	**<0.001**	0.25

Notes.

(a) male participants had higher overall score (mean ± s.d. = 14.58 ± 3.69) than female participants (13.15 ± 3.62)

(b) significant sex differences was found for DDF (p < .001, d = 0.62) and EOT (p < .001, d = 0.43) but not for the DIF subscale (p = .85, d = 0.02). These effects will not be further discussed as they are not of interest for the present research question. Also, these findings are replications of well-established findings and the interested reader is referred to Lane et al. [16] or Mattila et al. [15] for a thorough discussion.

(c) Main effect of scale is due to different minimum and maximum scores in the subscales, i.e. different number of items, and thus not further explored.

### Aging and the corpus callosum (analysis step 2)

**Fig 2:**
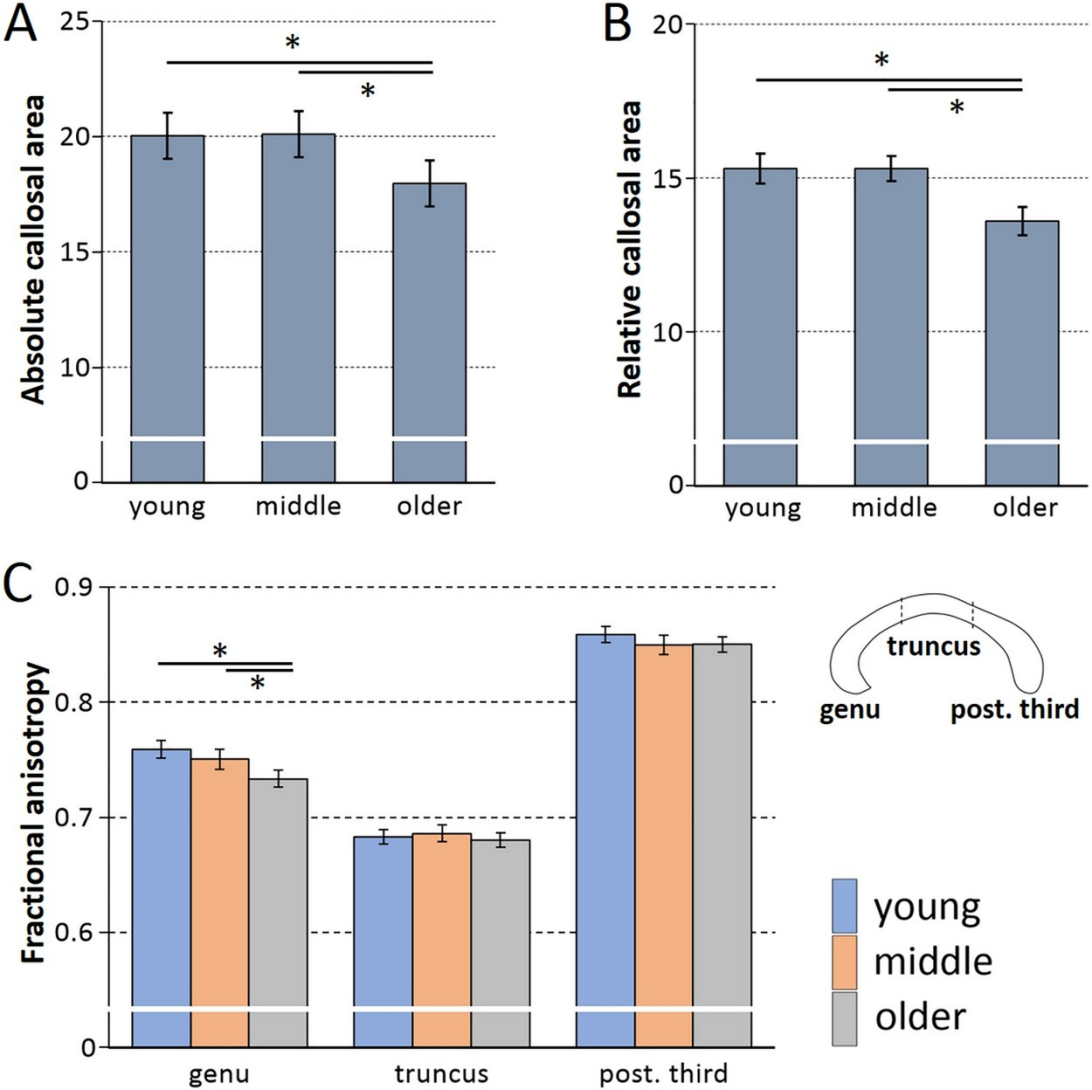
Aging and the corpus callosum. Mean absolute area (A), relative area (i.e., area/tIV^0.667^ ratio) (B), and fractional anisotropy, FA, (C) of the corpus callosum for the three age groups (error bars indicate *CI*_95%_). Panel (A) shows the main effect of age group, indicating a decrease in midsagittal area in the oldest compared to the two other groups. The y-axis provides the mean area measures across the three callosal subregions (in mm^2^*0.111). Panel (B) shows the main effect for relative area (as average across subregions) with again the oldest group differing from the two others. Panel (C) shows the age group FA means for each of the callosal subregions. Comparable to what was found for the two other parameters, also in the FA analysis the main effect of age was significant. However, an also significant age group by subregion interaction together with post-hoc testing revealed the age effect only to be significant in the genu. The sketch to the right of panel (C) illustrates the corpus callosum outline and the location of the three subregions (anterior: left). In all three panels asterisks (*) indicate significant post-hoc comparisons.

Aging-related changes in the corpus callosum were determined in three ANOVAs, one for each dependent variable (i.e., area, relative area, FA), with the between-subject factors age group and sex, as well as the repeated-measures factor subregion (genu, truncus, posterior third). The analyses revealed a main effect of age group for absolute and relative callosal area, and FA (*η*^*2*^ between .004 and 03, see Table 2). Across all three analyses, the same pattern emerged: the main effect was driven by a significant reduction in the older group compared with the other two groups, while no significant difference was found between the young and middle group (Fig 2). The effect size for absolute area was *d* = -0.03 (*p* = .82) comparing young and middle group, *d* = 0.69 (*p <* .001) comparing young and older group, and *d* = 0.71 (*p* < .001) comparing middle and older group. For the relative area, the effect sizes for young vs. middle group was *d* = -0.004 (*p* = .98), for young vs. older it was *d* = 0.75 (*p* < .001), and for middle vs. older it was *d* = 0.74 (*p* < .001). Finally, FA effect sizes were *d* = 0.16 (*p =* .24) for the differences between young and middle group, *d* = 0.44 (*p* = .004) for young vs. older group, and *d =* 0.26 (*p =* .057) comparing middle and older group.

**Table 2:** Aging and the corpus callosum. Results of the three analyses of variance with the three factors age group (young, middle, older), sex (male/female), and subregion (within factor: genu, truncus, posterior third) and using the dependent measure absolute area, relative area (absolute area/tIV^0.667^ ratio) and fractional anisotropy (FA), respectively.

			absolute area			relative area			fractional anisotropy		
Effect	df_effect_	df_error_	*F*	*p*	*η*^*2*^	*F*	*p*	*η*^*2*^	*F*	*p*	*η*^*2*^
Age Group	2	301	16.36	**<0.001**	0.03	18.49	**<0.001**	0.03	4.36	**0.014**	0.004
Age Group x Subregion	4	602	8.98	**<0.001**	<0.01	8.90	**<0.001**	<0.01	5.03	**<0.001**	<0.01
Age Group x Sex	2	301	1.98	0.14	<0.01	1.39	0.25	<0.01	1.68	0.19	<0.01
Age Group x Subregion x Sex	4	602	2.40	0.05	<0.01	2.01	0.09	<0.01	1.57	0.18	<0.01
Sex ^a^	1	301	9.49	**0.002**	0.01	4.41	**0.037**	0.004	7.77	**0.006**	0.004
Subregion ^b^	2	602	2455.30	**<0.001**	0.71	2415.36	**<0.001**	0.71	2511.71	**<0.001**	0.84
Subregion x Sex	2	602	1.92	0.15	<0.01	2.19	0.11	<0.01	0.54	0.59	<0.01

Notes.

(a) absolute area was larger in male than in females (*d* = -0.38); relative area was larger in females than in males (*d* = 0.25) thus replicating the findings of previous meta-analyses (e.g., ref. [66]). FA was higher in females than in males (d = 0.34). Sex differences will not be further discussed as they are well established and not relevant for the present research question.

(b) Subregional difference are inherent to the overall shape of the corpus callosum and the used subdivision schema, and are not discussed further.

Besides the main effect, also the interaction of age group and subregion was significant in all three analyses (all *η*^*2*^ < .01) indicating significant differences in the magnitude of the age effects between the subregions. For both absolute and relative size, post-hoc ANOVAs (factors: age group, sex) calculated separately for the subregions revealed a significant main effect of age group (all *F*(2,301)>6.92; all *p* < .001) in all subregions, whereby the strongest effect was always detected in the genu (absolute: *η*^*2*^ = .13, relative: *η*^*2*^ = .14) and the weakest in the posterior third subregion (absolute: *η*^*2*^ = .04, relative: *η*^*2*^ = .05). The age pattern was consistent across analyses: the older group differed significantly from the other two groups, while there was no difference between young and middle group. For FA, however, a significant age group effect was detected only in the genu subregion (see Fig 2C; *F*(2,301) = 12.87; *p* < .001, *η*^*2*^ = .08), with the older group showing reduced FA compared with the two other age groups (which did not differ from each other). No significant age group main effects were found in truncus (*F*(2,301)<1; p = .59; *η*^*2*^ < .01) and posterior third (*F*(2,301) = 1.99; *p* = .14; *η*^*2*^ = .01).

### Corpus callosum and emotional awareness (analysis step 3)

**Fig 3:**
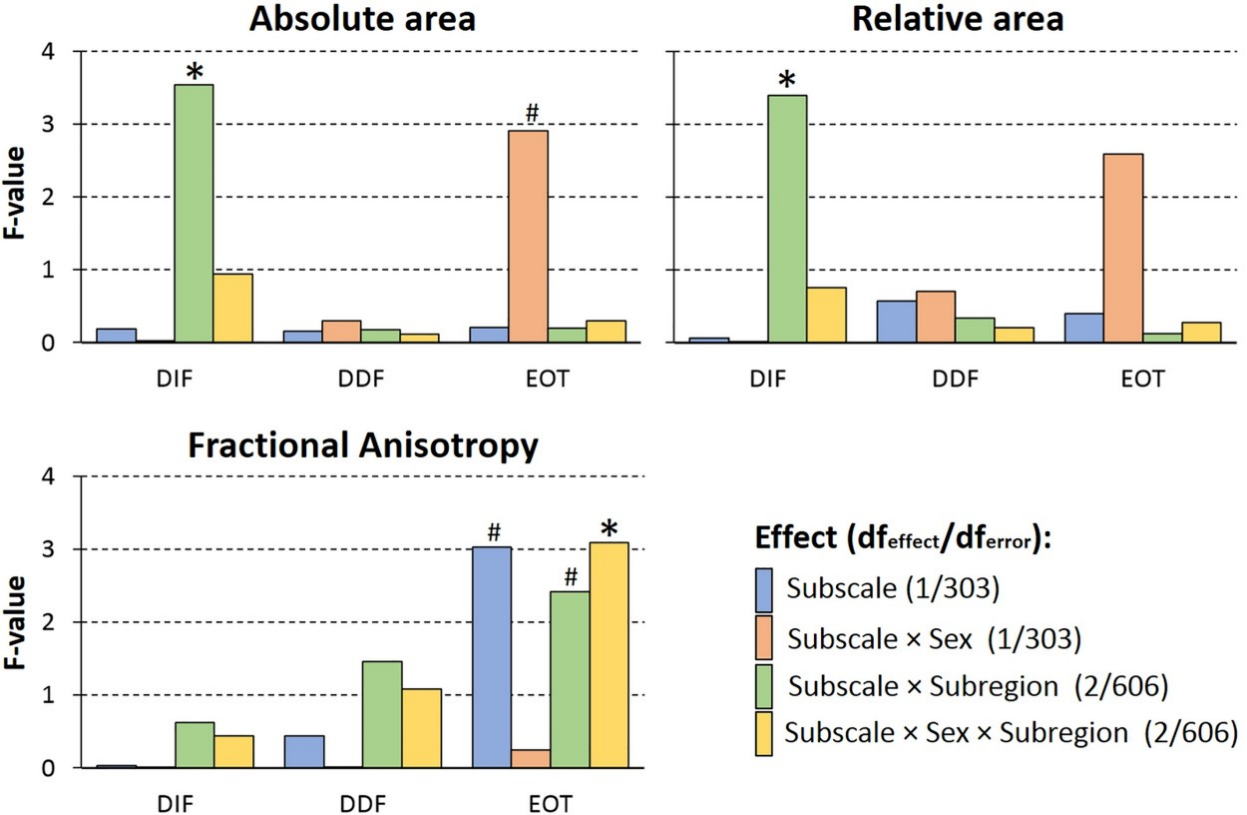
Callosal measures and emotional awareness. Bars represent the *F*-values for the effects of interest (i.e., main effect of subscale, interaction of subscale with sex and Region, respectively, and the three-way interaction) for the ANCOVAs conducted in the third analysis step. Results are shown separately for the three callosal parameters (panels) and the three TAS subscales (nested plots). Significant effects (*p* < .05) are indicated by asterisk (*), and trends (*p* < .10) by a number sign (#). Post-hoc analyses and effect size are reported in text. All effects common to the performed analyses (i.e., main effect of subregion and sex, as well as the subregion × sex interaction) are not depicted, but followed the same pattern as reported in analysis step 2.

To establish a direct association between each of the TAS subscales and the callosal parameters, we conducted separate analyses of covariance (ANCOVAs) with sex as between-subject factor, subregion as within-subject factor, and the respective subscale score as covariate. Callosal area, relative callosal area, or FA served as dependent variables in separate analyses. In all analyses, the main effect of subscale as well as interactions of subscale with other factors were effects of interest, indicating associations predicted by the callosal-relay model. As shown in Fig 3, neither for absolute or relative area, nor for FA an unconditional association with any of the three subscales was detected (all *η*^*2*^ < .01). Sensitivity power analyses indicated that for the main effect of subscale score and the interaction of subscale and sex, population effects of *η*_*min*_^*2*^ = .025 and larger can reliably be excluded. Additionally, to account for potential smaller subregional effects that are not optimally accounted for by the geometrical subdivision, we supplement the above analyses with a thickness analysis using the same statistical design predicting callosal thickness across the 60 measurement points. However, the regional thickness analysis did not yield any significant association (Details and results are provided in S1 Text and S1 Fig).

**Fig 4:**
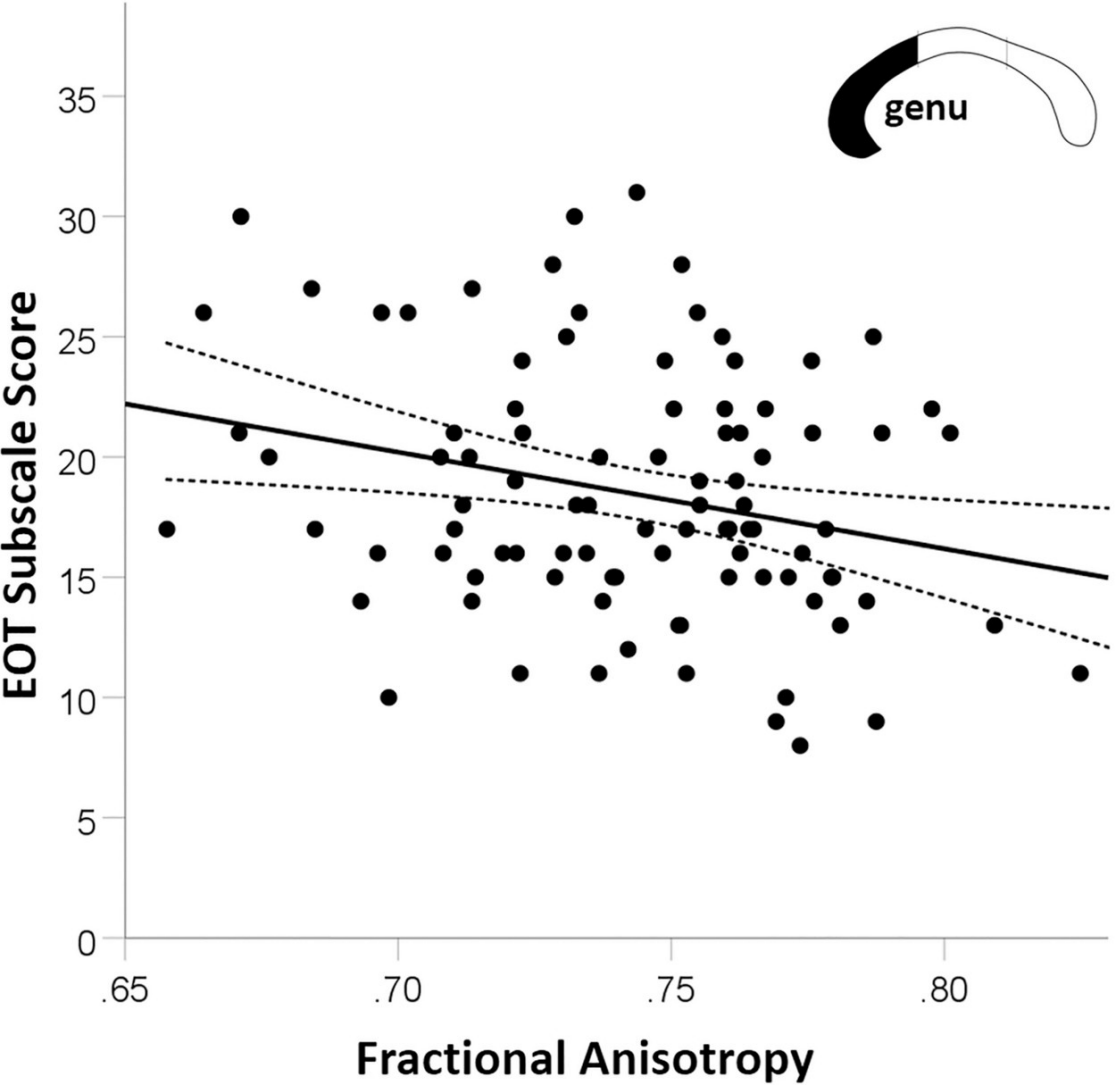
Association of EOT and genu FA. A significant negative correlation (*r =* -.259, *p =* .012) between FA in the genu and EOT in the male sample, was found to be the main factor underlying a significant three-way interaction of EOT, subregion, and sex. For no further callosal parameter, subregion, or subscale comparable effects were found. Details are presented in the Results section.

However, the ANCOVAs revealed several interaction effects. Firstly, considering absolute and relative callosal size, small but significant interaction effects of subscale and subregion were found for the DIF score (both *η*^*2*^ = .003). Post-hoc ANCOVAs (factor: sex, covariate: DIF) calculated separately for each subregion as well as for absolute and relative size, respectively, did not yield any significant main effect of DIF (all *F*(1,303)<1.604; all *p*>.21; *η*^*2*^ < .005). Thus, the above interactions were driven by difference in the correlation of subscale and callosal size between regions, but are not reflecting significant association of DIF with callosal measures. Secondly, for FA measures, the interaction of EOT, subregion, and sex reached significance (*η*^*2*^ = .01). Post-hoc ANCOVAs (factor: region, covariate: EOT) calculated separately for the two sexes revealed that the EOT by subregion interaction was significant in male (*F*(2,184) = 4.35; *p* = .014; *η*^*2*^ = .026) but not in female participants (*F*(2,422)<1; *p* = .67; *η*^*2*^ < .001), together driving the three way interaction. Follow-up analyses of the EOT by subregion interaction in male participants revealed a significant negative EOT-FA correlation in the genu (*r =* -.259, *p =* .012; Fig 4), but not in the two other subregions (truncus: *r =* -.148, *p =* .15; posterior third: *r =* .092, *p =* .38). Sensitivity power analysis for all interaction effects of the respective subscale and subregion as well as for the three-way interaction including sex, indicated sufficient test power for all effects larger than η_min_^2^ = .01.

### Aging, emotional awareness, and the corpus callosum (analysis step 4)

**Fig 5:**
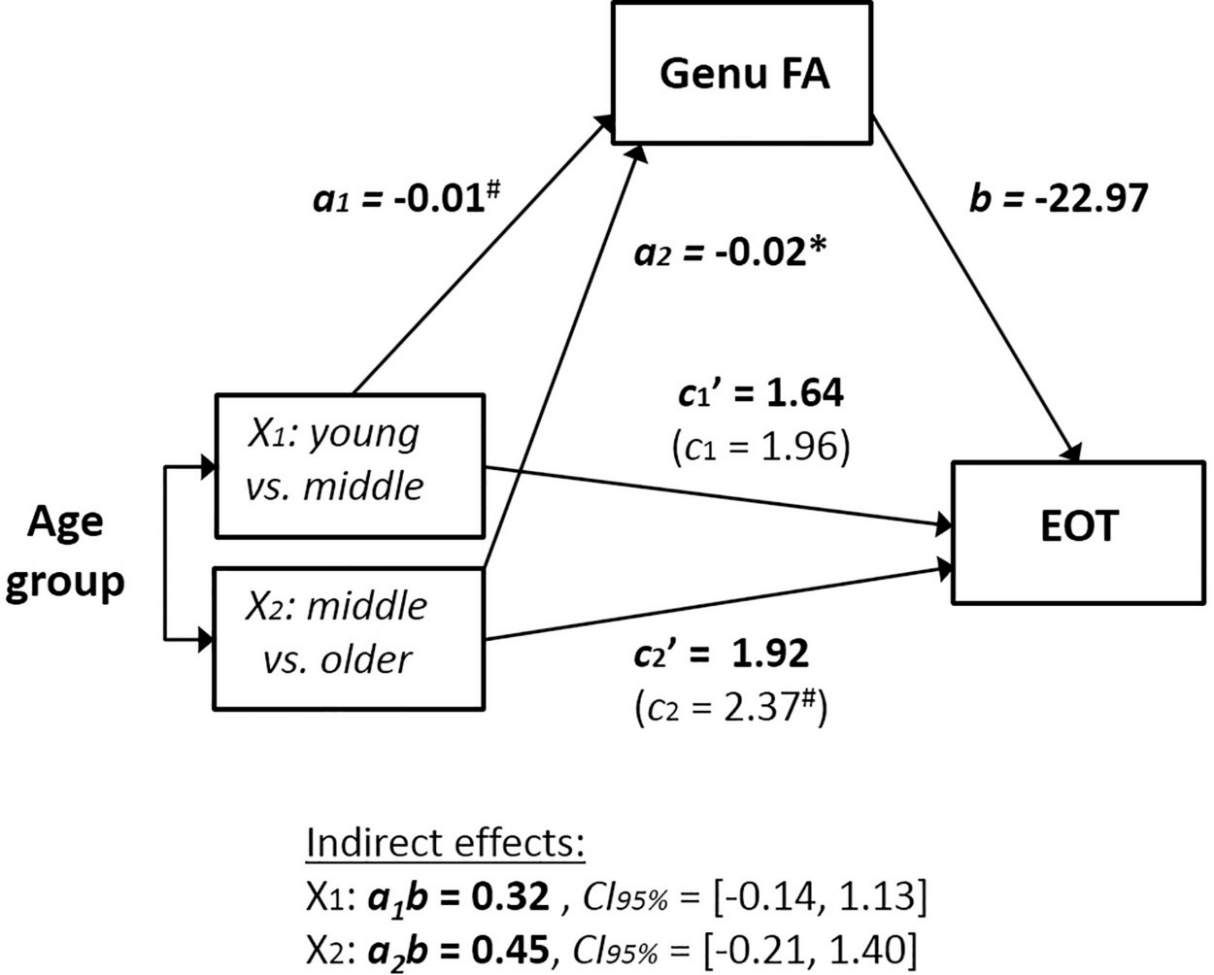
Mediation analysis. Estimated callosal mediation model for the prediction of EOT by age group and considering genu FA as mediator. Sequential coding was utilized for Age group, resulting in two predictors contrasting the young and the middle age group (X_1_) and the middle age with the older age group (X_2_), respectively. Parameter *a* represents the unstandardized regression weight for Age group prediction the mediator variable Genu FA, parameter *b* represents the regression weight for the mediator predicting the dependent variable EOT, and parameter *c*’ represent the direct effect of Age group on EOT. Parameter *c* represents the prediction without considering Genu FA. The indirect effect estimates are noted as *a*_*1*_*b* and *a*_*2*_*b*, respectively. Subscript numbers 1 and 2 of the path regression weights (a, c, and c’) indicate on which of the two Age group predictors the estimation was based. Significant effects (*p* < .05) are indicated by asterisk (*), and trends (*p* < .10) by a number sign (#).

Based on analysis steps 1 to 3, we restricted the mediation analysis (step 4) to genu FA and EOT in the male subsample. The mediation analysis was set-up with Age group as an independent variable. Since the factor Age group has three levels, two predictors were required, which were formed using sequential coding contrasting young and middle aged group (predictor X_1_) and middle and older aged group (X_2_) [73]. Genu FA served as mediator variable, and EOT as dependent measure (see Fig 5). In line with analysis step 2, Age group predicted Genu FA as trend for X_1_ with *a*_1_ = -0.01 (standard error, *s*.*e*. = 0.01), *t*(91) = 1.72, *p* = 0.09, and significantly for X_2_ *a*_2_ = -0.02 (*s*.*e*. = 0.008), *t*(91) = -2.64, *p* < .01, indicating a pronounced reduction in genu FA in the older group compared to the two other groups (omnibus model: *F*(2,91) = 8.99, *p* < .001, *r*^2^ = 0.17). The prediction of EOT by Genu FA was not significant, with *b* = -22.97 (s.e. = 16.68), *t*(90) = -1.38, *p* = 0.17. The direct effect of age group on EOT was significant in the omnibus test (*F*(2,90) = 3.23, *p* = .044; *r*^2^ = .06), reflecting the age differences in EOT as found in a analysis step 1. However, considering X_1_ and X_2_ separately, the direct effect was neither significant for X_1_ (*c*_1_’ = 1.64, *s*.*e*. = 1.32, *t*(90) = 1.24, *p* = .22) nor X_2_ (*c*_2_’ = -1.91, *s*.*e*. = 1.23, *t*(90) = 1.55, *p* = .12). The indirect effect was estimated for X_1_ (young-middle) as *a*_1_*b = 0.32 (*s*.*e*. = 0.33) with a CI_95%_ = [-0.14, 1.13] and effect size of *ab*_*ps*_ = 0.06, and for X_2_ (middle-older) as *a*_2_*b = 0.45 (*s*.*e*, = 0.41), with CI_95%_ = [-0.21, 1.40] and *ab*_*ps*_ = 0.09. Thus, both confidence intervals included zero, indicating the mediation effect of genu FA not to be significant. This was additionally confirmed by the Sobel Aroian test which yielded for X_1_ *z* = 0.98 with *p* = .33, and for X_2_ *z* = 1.16 with *p* = .25. Sensitivity power analysis was conceptualised as the minimal detectable (with *1-β* = .80) reduction in explained variance of the direct effect due to the introduction of the indirect effect to the prediction. A partial *R*_*min*_^*2*^ = .062 was determined (calculated using the degrees of freedom of the most complex model and expecting a one-sided test, i.e. a reduction).

In addition to the mediation analysis, we also calculated an ANCOVA (including the factors age group, sex, and the covariate EOT) to evaluate how introducing age changes the effects found in analysis step 3. The analysis neither revealed a significant main effect of EOT (*F*(1,295)<1; *p* = .34; *η*^*2*^ < .01) nor a significant interaction of age group and EOT (*F*(2,295)<1; *p* = .71; *η*^*2*^ < .01). Also, these effects were not modulated by sex; neither the sex by EOT (*F*(1,295)<1; *p* = .37; *η*^*2*^ < .01) nor the three-way interaction of age group, sex, and EOT (*F*(2,295)<1; *p* = .85; *η*^*2*^ < .01) were significant. Sensitivity power analyses indicates a detectable population effect of *η*_*min*_^2^ = .031 for the interactions including Age group, and of *η*_*min*_^2^ = .025 for the main effect of EOT and the interaction of EOT and sex.

## Discussion

Based on the callosal-relay model of emotional awareness and alexithymia [19, 20], the present study aimed to test the hypothesis that age-related decline of the corpus callosum significantly mediates the decrease in emotional awareness with age. For this purpose, it was first established that advancing age is associated with the predicted decline in emotional awareness as well as corpus callosum architecture. That is, the analysis of the TAS questionnaire revealed a selective age effect on the EOT subscale score, indicating a decrease in aspects of emotional awareness with age, which is in line with a series of previous studies [14–16, 18]. The average EOT was substantially higher in the older age group compared to the middle-aged and young group, while no comparable differences were found for the DIF/DDF subscales. Thus, the orientation or attention towards own emotions seem to be reduced in older age, while the clarity with which an individual can identify and describe emotions (when attended to) appears to be on a comparable level across age groups. This selective EOT effect confirms findings of two recent studies emphasizing that EOT is most strongly affected in older age [9, 74]. The EOT subscale, compared to DIF/DDF subscales, emphasizes the cognitive control aspect of emotional awareness; the tendency of an individual to voluntarily attend to and utilize emotions or actively suppress them [2, 62]. Thus, older age emotional awareness appears to be related to a reduction in these regulatory cognitive strategies, but not to changes in the identification of emotions.

Regarding the corpus callosum, the analyses indicate a reduction in interhemispheric connectivity with age across all callosal parameters. Midsagittal callosal size (absolute and relative area) and FA (in the genu) were found to be reduced in the older compared to the two younger groups. These age effects replicate previous findings of morphometric [44–46] and DTI studies [47, 48], and are likely reflecting an ongoing decline in fiber density or myelination of callosal axons with age [49–51]. Interestingly, no substantial differences were found between the young and middle age group in any of the three callosal parameters, suggesting that the predicted slow and continuous decline in the middle age group [44, 48, 60] is not sufficiently strong to be reflected in the age group mean differences to the young group.

In a third step, we sought to establish the predicted direct link between callosal connectivity and emotional awareness. Analyzing absolute and relative midsagittal area, and callosal thickness (see supplementary information S1 Text) we did not find any association with EOT, DIF, or DFF. Thus, the present study was not able to replicate the findings by Habib et al. [36] who had reported a negative association of TAS score with posterior callosal size. As the Habib et al. study examined multiple-sclerosis patients it can be speculated that the reported correlation might have reflected pathological processes rather than the effect of naturally occurring individual differences in callosal anatomy. At the same time, the test power of the present study allows to reliably exclude medium to large population effects (> 2.5% explained variance for main effect of test score, and>1% for regional differences interaction) so that the existences of any substantial association of callosal size measures and emotional awareness can be excluded.

However, analysing callosal FA, a negative correlation with EOT was detected in the genu of the corpus callosum, and restricted to male participants. Higher genu FA, which can be interpreted as “stronger” connectivity (i.e., higher axon density or stronger myelination, see e.g., [75]), was associated with a lower tendency for externalized thinking (i.e., lower EOT score). Or, referring to the items loading high on this subscale, a male participant with lower genu structural connectivity finds it less essential to attend to his emotions, prefers to talk about daily activities rather than feelings, and/or finds feelings less useful when solving problems. This association is in principle in accordance with the predictions of the callosal-relay model [19, 20]: lower callosal connectivity restricts the capacity for transfer of emotional information from the right hemisphere to the verbally competent left hemisphere. Seeing the left hemisphere as “the interpreter” [76, 77]–i.e., assuming only the left hemisphere forms a coherent interpretation of an event–right hemispheric emotional information would need to be accessed via the corpus callosum to be integrated in the interpretation of an event. Participants differing in callosal anatomy might thus differ in their readiness to utilize or attend to emotions, which could be reflected by the here observed negative correlation of EOT with genu FA: Once accessed via the corpus callosum, however, the identification and description of the emotion would not be affected by corpus callosum differences, as no correlation with DIF/DFF subscale was found.

Nevertheless, the found genu FA-EOT association (in males) demands further discussion as the correlation could merely reflect a temporal co-occurrence of a decline in emotional awareness (increase in EOT) and callosal FA reduction with age, without the two variables necessarily being functionally related. This suspicion is supported by two of our analyses: Firstly, the mediation analysis did not reveal a significant indirect effect via callosal FA when predicting EOT from Age group membership, and, secondly, repeating analysis step 3 while accounting for age group membership, did remove the effect of EOT in the male subsample. Thus, interpreted together with the results of analysis step 3 (i.e., no substantial association of callosal measures with DIF/DFF subscale) the present findings help specifying the callosal-relay model of emotional awareness. Surgical transection [21, 22, 24] or neuropathological alterations [7, 33, 36] of the corpus callosum evidently affect emotional awareness and may produce alexithymia-like symptoms. Normal occurring individual differences in callosal anatomy, however, appear not to be related to detectable differences in emotional awareness. Thus, as long as the corpus callosum is structurally intact, the exchange of emotional information from the right to the left hemisphere can take place. Nevertheless, measures of functional connectivity such as interhemispheric coherence in neuronal activity [37] or measures of quality/speed of hemispheric interaction [41, 43] appear better suited to capture relevant individual differences in the dynamic aspects of hemispheric cooperation.

Importantly, our results are also at odds with the hypothesis of a corpus callosum decline mediated age effect on EOT. Callosal decline can be expected to be accelerated in the older age group [44, 48, 51, 60] and in this add age-related variance to the individual difference so that the variance of callsoal FA values within the older group should be increased compared to the variance in the young and middle age groups. In turn, if callosal FA is relevant for EOT values this should also increase the variance of the EOT values and lead to stronger FA-EOT covariance in the older compared to the two other groups. However, comparable indirect mediation effects and the non-significant interaction in step 4 are not in line with this prediction. At the same time, test power of both analyses is sufficiently high to exclude substantial mediation (>6% explained variance) or interaction effects in the population (> 2.5–3% explained variance). Consequently, substantial age group differences in the FA-EOT association can be excluded, rendering a contribution of callosal decline to the observed age-related reduction in emotional awareness unlikely. Of note, as we only were able to utilize cross-sectional data, it cannot be decisively concluded that at least part of the observed variance in the data might be attributed to cohort rather than developmental effects. Thus, a final conclusion has to await replication with longitudinal data.

A limitation of the present approach might be seen in the fact that emotional awareness was assessed with a self-report questionnaire. Consequently “classical” self-report biases, such as social-desirability, cannot be fully excluded [78], and one might speculate that the age or sex groups might differ systematically in the susceptibility to these biases [79]. However, the TAS-20 questionnaire is the result of rigorous psychometric evaluation with respect to reliability and validity [2, 56, 57, 61] and has accordingly been repeatedly and successfully applied to assess emotional awareness also in non-clinical samples, including aging samples [15, 16, 62, 74]. Nevertheless, future studies might consider to additionally utilize experimental assessment (e.g., emotional Stroop task [2]) or measures of physiological interoception (e.g., heart beat perception [63]) to also include non-self report data into the analysis. Especially time critical paradigms using emotional stimuli appear promising to assess quality and speed of information transfer in context of emotional processing [20]. Finally, the TAS scores were in the normal range and below cut-offs for clinical alexithymia. Thus, the present findings cannot exclude that callosal alterations can be found in clinical alexithymia samples.

Taken together, the present study confirmed the expected cross-sectional decline in both callosal anatomy and emotional awareness with aging. However, we were not able to establish any substantial association of inter-individual difference in callosal anatomy and differences in emotional awareness. Thus, we were neither able to find support for the callosal-relay hypothesis of emotional awareness nor for a contribution of callosal deterioration to reduced emotional awareness with age.

## Supporting information

S1 Text Supporting analysis. Callosal thickness and emotional awareness.
(DOCX)

S1 Fig Results of supporting analysis. Visualising the main effect of subscale on callosal thickness for the three subscales. At each callosal segments, the direction and magnitude of the association is indicated by a circle. The size of the circle is proportional to *t*-value for the subscale predictor. Positive and negative associations (i.e., the sign of the regression β-weights of the respective predictor) are coded orange and blue, respectively. Light orange and light blue indicate non-significant associations, as for no segment a significant association was found (at a False-Discovery-Rate, FDR, of 0.05). Note: the outline represents the mean corpus callosum outline across all participants and with anterior corpus callosum on the left side of each panel. Vertical dashed lines indicate the callosal subdivision as implemented in the area and FA analyses.
(TIF)
