# Supplementary material for: Parallel but independent reduction of emotional awareness and corpus callosum connectivity in older age
Source: PLoS One. 2018 Dec 31;13(12):e0209915. doi: 10.1371/journal.pone.0209915 (PMC6312250; doi:10.1371/journal.pone.0209915)
Supplement: S1 Text — Callosal thickness and emotional awareness. (DOCX) [file pone.0209915.s001.docx]

***S1 Text: Callosal thickness and emotional awareness***

In the main analysis subregions of the corpus callosum were defined by the well-established geometric subdivision schema originally suggested by Witelson [1]. However, it might be argued that this subdivision approach is insensitive to more subtle variations in callosal morphology. Thus, to increase spatial sensitivity we supplemented the main structural analysis with a callosal thickness analysis, which assesses variations in thickness along the midsagittal cross-sectional surface of the corpus callosum [2].

*Material and methods*. The here employed analysis approach has been described elsewhere [3, 4] and is here only briefly summarized. Based on the extracted individual callosal masks (see section 2.4), callosal outlines were created by removing all non-border voxels from the callosal mask. The outline was then split into a ventral and dorsal part, whereby rostrum tip and the base of the splenium marked the separation points between ventral and dorsal outlines. The geometric midline between ventral and dorsal outline was calculated which was resampled into 60 equidistant support points. Orthogonal to the support points the distance between ventral and dorsal outline was determined, resulting in 60 regional thickness measures for each participant (in mm).

The statistical analysis of the thickness data was restricted to test the callosal-relay hypothesis of emotional awareness [5]; reflecting analysis step 3 of the main analysis. Analyses were set up separately for each TAS Subscales, and using an analysis of covariance (ANCOVAs) design: Sex was used as between-subject factor and the respective TAS subscale (DIF, DDF, EOT) served as covariate, whereby the interaction of covariate and Sex was included as additional predictor. Analysis were conducted using the “fitlm” MATLAB function and significance level were adjusted to yield a False-Discovery-Rate (FDR) of 0.05 for each analysis separately.

*Results*. For none of the three parameters a statistical significant association with callosal thickness was detected. The findings are visualised in *S2 Fig*, illustrating the main effect of Subscale. At each callosal segments, the direction and magnitude of the association is indicated by a circle. The size of the circle is proportional to *t*-value for the Subscale predictor. Positive and negative associations (i.e., the sign of the regression β-weights of the respective predictor) are coded orange and blue, respectively. Light orange and light blue indicate non-significant associations, as for no segment a significant association was found (at a False-Discovery-Rate, FDR, of 0.05).

*References*

1. Witelson SF. Hand and sex differences in the isthmus and genu of the human corpus callosum. Brain. 1989;112(3):799-835.

2. Luders E, Toga AW, Thompson PM. Why size matters: differences in brain volume account for apparent sex differences in callosal anatomy: the sexual dimorphism of the corpus callosum. Neuroimage. 2014;84:820-4.

3. Westerhausen R, Fjell AM, Krogsrud SK, Rohani DA, Skranes JS, Håberg AK, et al. Selective increase in posterior corpus callosum thickness between the age of 4 and 11 years. NeuroImage. 2016;139:17-25.

4. Westerhausen R, Friesen C-M, Rohani DA, Krogsrud SK, Tamnes CK, Skranes JS, et al. The corpus callosum as anatomical marker of intelligence? A critical examination in a large-scale developmental study. Brain Structure and Function. 2018;223(1):285-96.

5. Wingbermühle E, Theunissen H, Verhoeven W, Kessels RP, Egger JI. The neurocognition of alexithymia: evidence from neuropsychological and neuroimaging studies. Acta Neuropsychiatrica. 2012;24(2):67-80.
