# Supplementary figures and images for: Parallel but independent reduction of emotional awareness and corpus callosum connectivity in older age
Source: PLoS One. 2018 Dec 31;13(12):e0209915. doi: 10.1371/journal.pone.0209915 (PMC6312250; doi:10.1371/journal.pone.0209915)

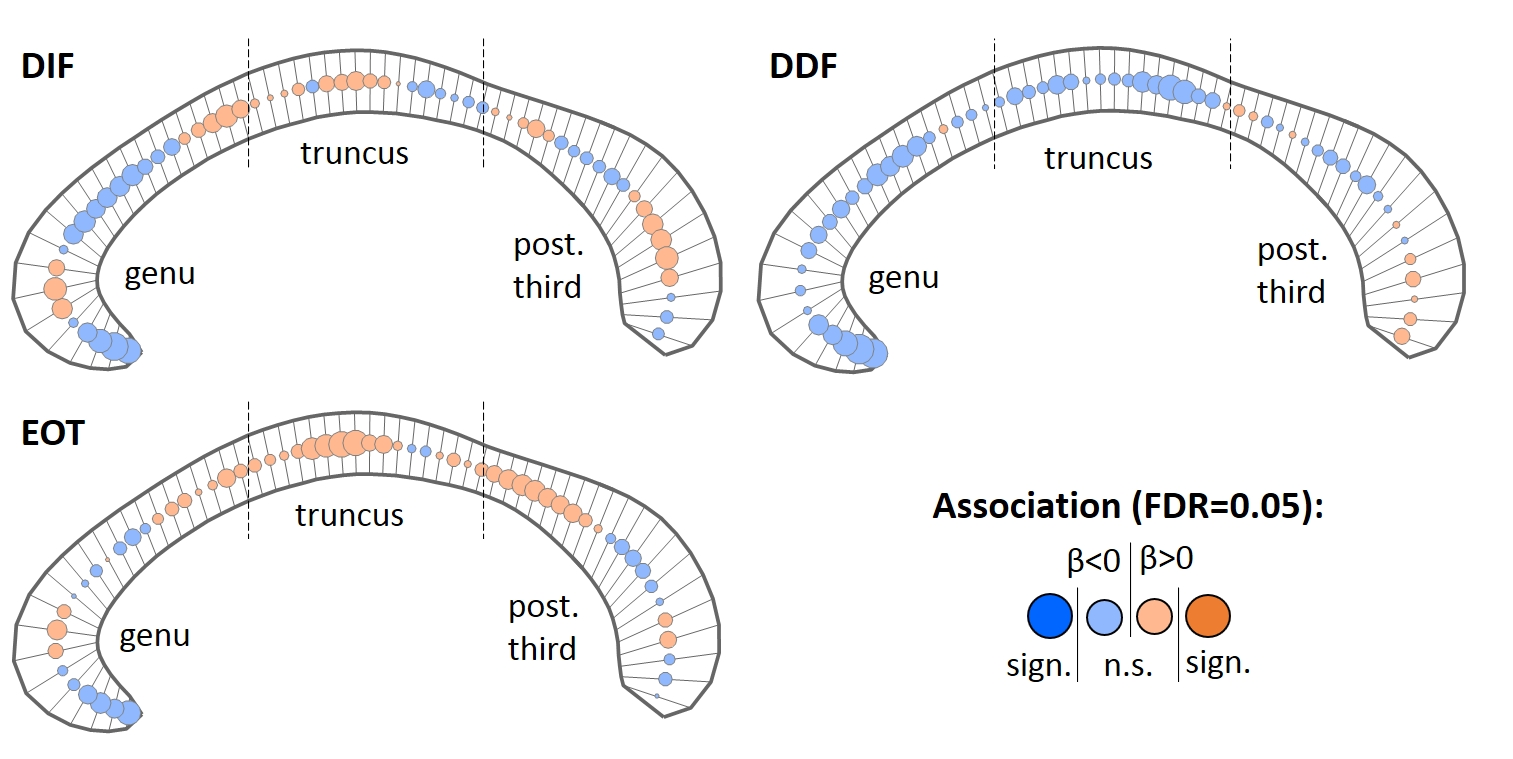

Supplement: S1 Fig — Visualising the main effect of subscale on callosal thickness for the three subscales. At each callosal segments, the direction and magnitude of the association is indicated by a circle. The size of the circle is proportional to t-value for the subscale predictor. Positive and negative associations (i.e., the sign of the regression β-weights of the respective predictor) are coded orange and blue, respectively. Light orange and light blue indicate non-significant associations, as for no segment a significant association was found (at a False-Discovery-Rate, FDR, of 0.05). Note: the outline represents the mean corpus callosum outline across all participants and with anterior corpus callosum on the left side of each panel. Vertical dashed lines indicate the callosal subdivision as implemented in the area and FA analyses. (TIF) [file pone.0209915.s002.tif]
